# Characteristics of Hearing Loss in Patients with *COL2A1* Gene Variants (Sticker Syndrome Type 1)

**DOI:** 10.1007/s12070-025-05638-7

**Published:** 2025-06-12

**Authors:** María Fábrega-Torrano, Rocío González-Aguado, Esther Onecha, Carmelo Morales-Angulo

**Affiliations:** 1 Facultad de Medicina, Universidad de Cantabria, Santander, Spain; 2 Department of Otolaryngology, Hospital Universitario Marqués de Valdecilla, Santander, Cantabria Spain; 3 Department of Genetics, Hospital Universitario Marqués de Valdecilla, Santander, Cantabria Spain; 4 Cellular Signaling and Therapeutic Targets in Cancer, Institute for Research Marqués de Valdecilla (IDIVAL), 39011 Santander, Spain; 5 Cell Cycle, Stem Cell Fate and Cancer Laboratory, Institute for Research Marqués de Valdecilla (IDIVAL), 39011 Santander, Spain

**Keywords:** *COL2A1*, Stickler Syndrome, Autosomal dominant, Genetic hearing loss, Sensorineural hearing loss

## Abstract

Stickler syndrome type 1 (STL1) is an autosomal dominant genetic disorder that affects connective tissue with highly heterogeneous clinical manifestations, primarily at the ocular, orofacial, skeletal, and auditory levels. The aim of our study was to determine the variability of the auditory phenotype associated with pathogenic variants of the *COL2A1* gene and its impact on long-term treatment and follow-up. A retrospective observational study was conducted on patients with a confirmed diagnosis of STL1 treated between 2018 and 2024 at the Otorhinolaryngology Department of the Marqués de Valdecilla University Hospital. The electronic medical records of the patients included in the study were reviewed to gather the following information: family history, age, sex, genetic variant of STL1, audiological test data, associated clinical manifestations, radiological tests, and treatment. Among the 8 patients included in the study, 6 (75%) exhibited hearing loss, with severity ranging from mild to profound. Of these, 4 (66.7%) had sensorineural hearing loss, 1 (16.7%) had conductive hearing loss, and 1 (16.7%) had a mixed type. The 4 patients with sensorineural hearing loss required hearing aid fitting, and one of them also needed a cochlear implant, showing significant improvement in language comprehension. Another patient required a mastoidectomy due to a cholesteatoma. The results of our study suggest that hearing loss in patients with STL1 is a common finding, predominantly sensorineural in nature and of variable intensity.

**Supplementary Information:**

The online version contains supplementary material available at 10.1007/s12070-025-05638-7.

## Introduction

**Fig. 1:**
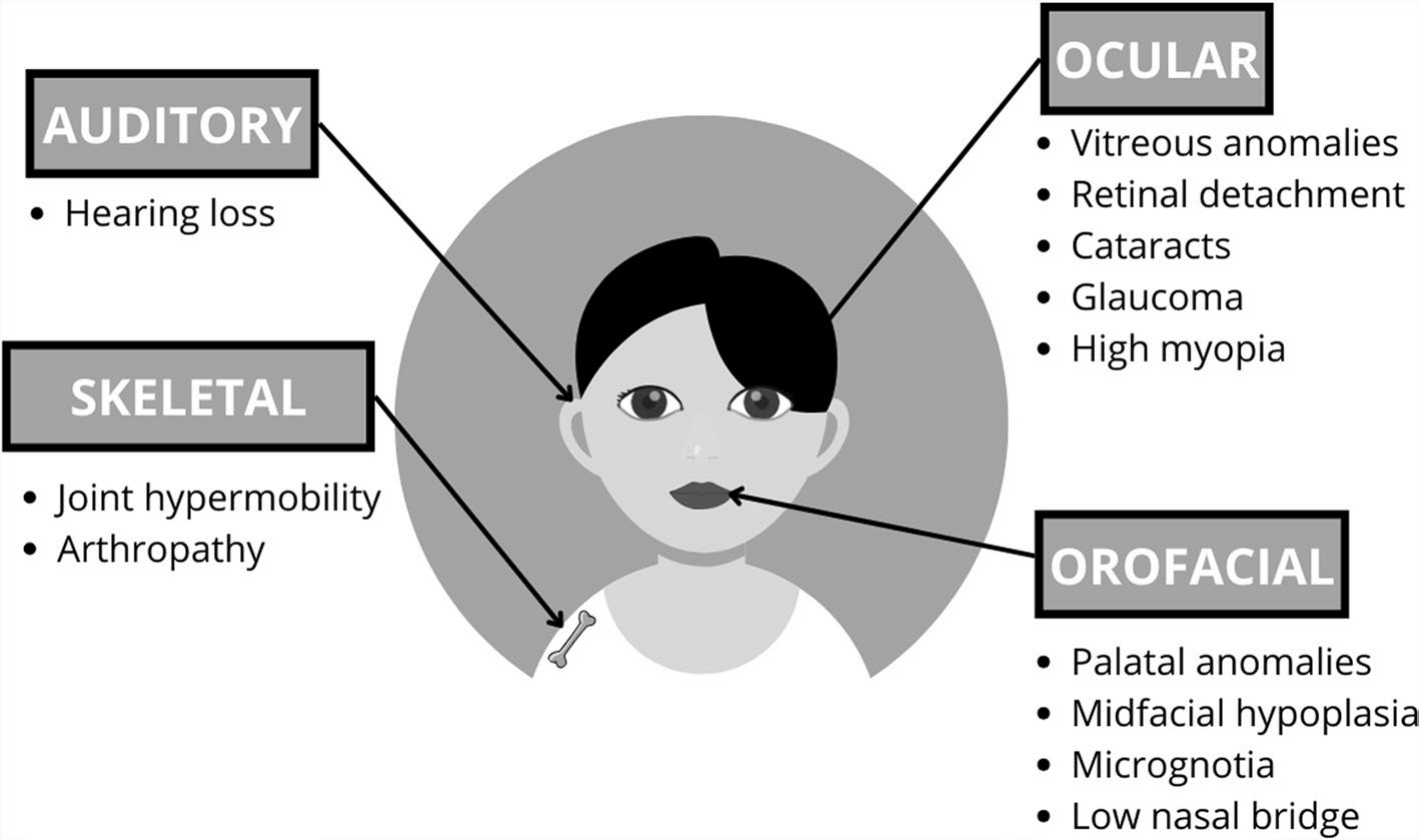
Clinical (**a**) and ocular (**b**) manifestations of Stickler Syndrome

Stickler Syndrome (SS), or progressive hereditary arthro-ophthalmopathy (ORPHA828), is a connective tissue disorder characterized by ocular, skeletal, orofacial, and auditory abnormalities [1, 2]. Typical features include vitreoretinal degeneration, high-grade myopia, retinal detachment, glaucoma, cataracts, premature osteoarthritis, midfacial hypoplasia, cleft palate, and hearing loss [1, 2] (Fig. 1). Moreover, SS is the most common cause of retinal detachment in childhood and familial retinal detachment, which can lead to blindness at an early age [3].

SS presents considerable phenotypic heterogeneity due to the wide variety of genes and mutations involved in the encoding and assembly of type II, IX, and XI collagen. These mutations can be inherited in either a dominant (AD) or recessive (AR) manner [4–6].

The most common form is Stickler Syndrome type 1 (STL1), which follows an AD inheritance pattern and is caused by heterozygous variants that result in loss of function of the *COL2A1* gene, leading to haploinsufficiency [3]. In these patients, the most prominent manifestations are ocular, specifically high-grade myopia and vitreous membrane, along with hearing loss [1, 2, 4, 7, 8]. Because of these features, patients with STL1 are often diagnosed in Ophthalmology and Otolaryngology clinics. Other manifestations of STL1 include palatal anomalies (cleft palate, submucous cleft, high-arched palate) and osteoarticular issues, which occur in 41% of patients before age 10 and in 90% of those over 40 years of age [9].

The COL2A1 gene encodes the α-1 chain of type II procollagen, which consists of three identical chains folded into a helical structure. Mature type II collagen is synthesized after the secretion of procollagen into the extracellular space, followed by cleavage of the NH2 and COOH propeptides [10, 11]. This processed collagen forms a fibrillar network, covalently linked to the extracellular matrix, to provide tensile strength to the tissues in which it is found [12, 13]. Mutations in the *COL2A1* gene associated with the development of STL1 are primarily loss-of-function mutations, often caused by premature stop codons that lead to messenger RNA (mRNA) degradation [8]. However, glycine substitutions that disrupt the triple helical structure of the collagen chain can also cause STL1 and appear to be associated with a higher risk of hearing loss [1].

Type II collagen is the main component of hyaline articular cartilage and is also present in vitreous structures and the inner ear [14], specifically in the inner and outer hair cells and the tectorial membrane [15]. This localization of type II collagen explains why the most significant manifestations of STL1 are those previously mentioned. Furthermore, due to the considerable phenotypic variability observed both within and between families of patients with STL1, it is impossible to predict which patients will develop hearing loss.

On the other hand, the auditory phenotype has been scarcely studied. As a result, the prevalence and characteristics of hearing loss in patients with STL1 are not well defined [2].

The aim of our study was to determine the frequency and characteristics of the auditory phenotype based on genetic variants in the *COL2A1* gene and its impact on long-term treatment and follow-up.

## Patients and Methods

### Study Design

A retrospective observational study was conducted on patients with variants in the *COL2A1* gene who were seen between January 2018 and December 2024 at the Marqués de Valdecilla University Hospital (HUMV) in Santander, a tertiary referral hospital for the Autonomous Community of Cantabria, serving approximately 570,000 inhabitants.

### Variables

From the medical records, we collected family history, sex, whether the patient developed hearing loss or not, and, in case of hearing loss, the age of onset, type (sensorineural, conductive, or mixed), laterality (unilateral or bilateral, symmetric or asymmetric), and progression (stable, fluctuating, or progressive).

We also recorded associated otologic disorders (tinnitus, vertigo, otalgia, or otorrhea). Additionally, findings related to systemic involvement were noted.

The degree of hearing loss on pure-tone audiometry, conducted in a soundproof booth, was classified according to the criteria established by the American Speech-Language-Hearing Association [16]:

- Normal hearing: thresholds between 10 and 15 dB.
- Minimal hearing loss: thresholds between 16 and 25 dB.
- Mild hearing loss: thresholds between 26 and 40 dB.
- Moderate hearing loss: thresholds between 41 and 55 dB.
- Moderate-severe hearing loss: thresholds between 56 and 70 dB.
- Severe hearing loss: thresholds between 71 and 90 dB.
- Profound hearing loss: thresholds greater than 91 dB.

The pattern of hearing loss across the frequency spectrum evaluated during tonal audiometry was classified as:

- Descending: hearing loss was greater in the high frequencies than in the low frequencies.
- Flat: hearing loss was relatively uniform across all frequencies.
- Ascending: hearing loss was greater in the low frequencies than in the high frequencies.
- Mid-frequency: hearing loss was more pronounced in the mid frequencies, while low and high frequencies may be less affected.

Progression was assessed by comparing serial audiograms over time in patients for whom such data were available in the medical records, calculating the annual hearing loss in decibels (dB).

The medical records also included whether patients had undergone brainstem auditory evoked potentials (BAEP), otoacoustic emissions (OAE), vestibular testing, temporal bone computed tomography (CT), and magnetic resonance imaging (MRI) of the pontocerebellar angles and/or posterior fossa.

### Genetic Analysis

DNA sequencing techniques, both Sanger and Next Generation Sequencing (NGS), were employed. Sanger sequencing was used for patients with a direct suspicion of SS, while NGS was employed for those with suspected genetic hearing loss but without an initial SS suspicion.

For the latter group, NGS was carried out using a standard workflow comprising both laboratory and bioinformatic steps aimed at the rapid and accurate analysis of genomic DNA. The process included:

1. Sample Preparation—Genomic DNA was extracted from peripheral blood samples.
2. Library Preparation—DNA was fragmented and ligated with sequencing adapters.
3. Target Enrichment—A custom gene panel associated with hearing loss, including *COL2A1* (see Supplementary Material), was used. Target regions were captured using Sure Select RNA probes (Agilent Technologies, Inc., San Diego, CA, USA).
4. Sequencing—Libraries were sequenced on the Illumina MiSeq platform (Illumina, San Diego, CA, USA).
5. Data Processing—Base calling and demultiplexing were performed to generate raw sequence data.
6. Alignment and Variant Calling—Reads were aligned to the hg19 reference genome using BWA-MEM, and variant calling was conducted using SAMtools and associated tools.
7. Variant Annotation and Interpretation—Identified variants were annotated using multiple databases, including Ensembl, CCDS, RefSeq, Pfam, dbSNP, gnomAD, 1000 Genomes, ESP6500, ExAC, ClinVar, HGMD Professional, OMIM, Orphanet, and GeneReviews.

Variant classification was performed according to five categories: pathogenic, likely pathogenic (LP), variant of uncertain significance (VUS), likely benign (LB), and benign (B), following the ACMG guidelines and expert specifications for interpreting genetic variants in hearing loss.

### Data Collection

Patient data was obtained from electronic medical records, including reports of complementary tests. An Excel database was created with all the variables described above. Identifying patient information was dissociated and encoded using an internal code to prevent direct identification of the patients. Only data relevant to the study’s objective was included. A descriptive statistical study was performed on the cases included in the study.

### Ethical Aspects

The study was conducted following Good Clinical Practice guidelines, in accordance with the principles of the Declaration of Helsinki and Spanish legislation. The study was approved by the Cantabria Ethics Committee for Research with Medicinal Products and Health Products (CEIM), under internal code 2024.425 (dated January 24, 2025).

## Results

During the 7 year study period, variants in the *COL2A1* gene were detected in 8 patients (6 probands and 2 familial cases), of whom 5 were women (62.5%) and 3 were men (37.5%), with ages ranging from 1 to 84 years at the time of diagnosis. The demographic, clinical characteristics, allelic variants, and treatment are summarized in Table 1. In our study, 5 pathogenic or likely pathogenic variants of the *COL2A1* gene and one VUS (F6/P1) were found.

**Table 1:** Demographic, clinical, genetic characteristics, and treatment of patients with STL1

Patient ID. age/gender	Reason for genetic study	Family history	Hearing loss, type and age at diagnosis	Another associated condition	Genetic variant (*COL2A1*)	Treatment
F1/P1, 1/M	Craniofacial features suspicious for SS	Yes	Normacusis transient CHL	Myopia, Palatal cleft	c.1468_1475delinsT	NA
F1/P2, 50/M	Family segregation study	Yes	Moderate CHL (0–10)	Palatal cleft	c.1468_1475delinsT	Mastoidectomy, Tympanoplasty, Contralateral drainage
F1/P3, 84/Fe	Family segregation study	Yes	Normacusis	Myopia, Vitreous abnormalities	c.1468_1475delinsT	NA
F2/P1, 60/Fe	Hearing loss	No	Moderate/Severe SNHL, (high frequencies)(50)	Bilateral RD, Osteoarticular manifestations	C816 + 1G > A, mutation in the *COCH* gene associated	HA
F3/P1, 50/M	Hearing loss	Yes	Profound SNHL (20–30)	Cataracts, Palatal cleft, Osteoarticular manifestations	c.3583G > T, p.Glu119S	HA, CI
F4/P1, 56/Fe	Hearing loss	Yes	SNHL (RE: flat, LE: high frequencies), RE: Mild and, LE: Profound, (40–50)	No	c.1833 + 1G > A, mutation in the *COCH* gene associated	HA
F5/P1, 58/Fe	Family segregation study	Yes	Moderate/Severre SNHL (Flat curve), (Age at diagnosis unknown)	Glaucoma, Osteoarticular manifestations	c.1783delG, p.Ala595Leufs*34	HA
F6/, 31/Fe	Hearing loss	Yes	Mix HL (RE: Mild, LE: Severe) (Age at diagnosis unknown)	No	c.3111 + 5G > A (VUS)	No treatment

F, Family; P1, Proband; M, Male; Fe, Female; SS, Stickler síndrome; AD, Autosomal dominant; HL, Hearing loss; RE, right ear; LE, Left ear; SNHL, Sensorineural hearing loss; CHL, Conductive hearing loss; RD, Retinal detachment; OMC, Cholesteatomatous otitis media; VUS, Variant of uncertain significance; HA, Hearing aids; CI, Cochlear implant; NA, Not applicable

The reasons for genetic testing were suspected hereditary hearing loss in 4 patients (50%), craniofacial features in 1 patient (F1/P1), and participation in a family segregation study in 3 cases (F1/P2, F1/P3, and F5/P1), as patient F5/P1 had 3 children and 1 grandchild diagnosed with SS in another Autonomous Community.

**Fig. 2:**
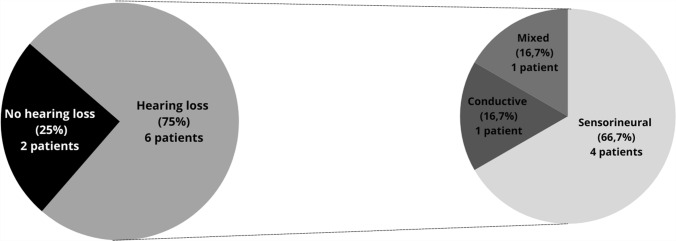
Prevalence and type of hearing impairment in our series

**Fig. 3:**
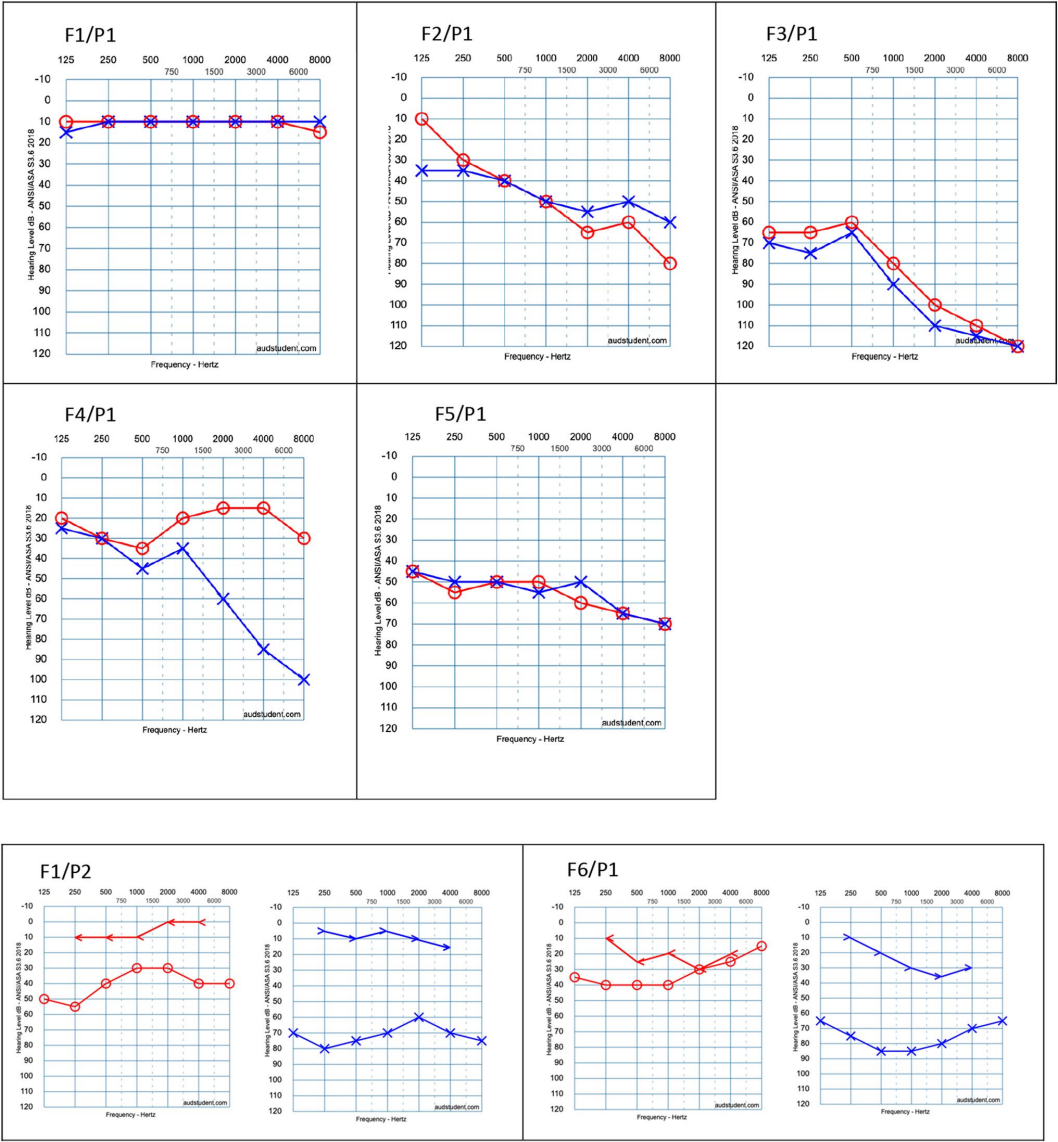
Audiograms of the patients included in the study. Red: right ear. Blue: left ear. In F1/P1, the air conduction (AC) and normal hearing are represented. In F2/P1, F3/P1, F4/P1, and F5/P5, AC is represented and reflects sensorineural hearing loss. In F1/P2 and F6/P1, both air and bone conduction (BC) are represented, with the former showing a conductive hearing loss and the latter a mixed hearing loss

Hearing loss was observed in 6 out of 8 patients (75%), with 4 of them having sensorineural hearing loss (SNHL) (66.7%), 1 with conductive hearing loss (16.7%), and 1 with mixed hearing loss (16.7%), affecting both ears in all cases (Fig. 2 and Table 1). In 3 patients, the hearing loss was asymmetric when comparing both ears. The severity ranged from mild to severe/profound hearing loss as shown in Fig. 3. Additionally, 2 patients showed significant progression of hearing loss compared to the physiological age-related auditory deterioration. As for vestibular symptoms, only 1 patient (F2P1) presented with a condition compatible with benign paroxysmal positional vertigo (BPPV). Another patient (F4P1) was evaluated for dizziness, presenting normal V-HIT and VNG results.

Moreover, 6 out of 8 patients exhibited other typical STL1 manifestations due to collagen involvement at other levels. The associated pathology varied between patients and included ocular, joint, and palatal manifestations (Table 1). A palatal defect was present in 37.5% (3/8) of the studied population. Of these 3 patients with a palatal malformation, 2 had hearing loss, one of which was conductive and the other sensorineural (Table 1). Four patients underwent imaging tests (CT/MRI), with normal results in 2, 1 showing middle ear pathology consistent with cholesteatoma (patient F1/P2), and another (F6P1) presenting endolymphatic sac dilation in the left ear on both CT and MRI.

Regarding treatment, the 4 patients with SNHL required hearing aids, and one of them later needed a cochlear implant (CI), with a good response in terms of speech comprehension (Table 1). Additionally, a patient with a cleft palate (F2/P2) and cholesteatomatous otitis media required surgical intervention, including mastoidectomy with tympanoplasty and contralateral transtympanic drainage.

## Discussion

We retrospectively analyzed all patients diagnosed with *COL2A1* gene mutations over a 7 year period. In contrast to previous reports, which have described hearing loss prevalence in SS patients ranging from 17.2 to 74% (Table 2) [2, 6, 8, 17, 18], our cohort showed a relatively high prevalence (75%). This could be partially explained by the fact that in half of our probands, hearing loss was the primary reason for genetic testing, without prior suspicion of SS. Age distribution may also contribute to prevalence differences across studies, with younger cohorts tending to show lower rates of hearing loss. As observed previously, SNHL was the predominant subtype [2, 8].

**Table 2:** Auditory manifestations in patients with STL1 literature review

Author	Year	Country	Systematic review or case series	Number of cases included	Hearing loss (%)	Type of hearing loss (%)
Acke et al. [2]	2012	Belgium	Systematic review	224	52.2	SN: 66.2%
						T: 13.4%
						M: 19.92%
Hoornaert et al. [6]	2010	Belgium	Case series	100	74	SN: 68.9%
						T: 31.1%
Terhal et al. [25]	2015	Netherlands	Case series	93	17.2	ND
Bath et al. [13]	2021	United States	Case series (pediatric)	16	25	SN: 25%
						T: 25%
						M: 25%
						NA: 25%
Choi et al. [26]	2021	Korea	Case series	30	30	ND

SN, Sensorineural; T, Transmission; M, Mixed; NA, Not available; ND, Not determined

Although SNHL in STL1 has been classically described as bilateral, high-frequency, mild, and relatively non-progressive [1, 2, 19], our study demonstrated substantial variability. Severity ranged from mild to profound, and in some cases, progression exceeded age-related expectations (Table 1 and Fig. 3). These findings underscore the broad phenotypic heterogeneity of STL1.

Patient F4/P1, a 56 year-old woman with profound SNHL, carried a pathogenic *COL2A1* variant (NM_001844.5:c.1833 + 1G > A) [20]. However, she also carried a *COCH* gene mutation known to cause progressive SNHL, and presented no other STL1-related features, raising questions about the true etiologic origin of her hearing loss.

In another case (F5/P1), a novel *COL2A1* variant (c.1783delG, p.Ala595Leufs*34) classified as likely pathogenic was identified during genetic work-up for hearing loss. The patient also had osteoarticular symptoms and glaucoma, supporting the variant’s clinical relevance.

All 4 patients with SNHL required hearing aids; in one case (F3/P1), a CI was necessary and resulted in excellent speech discrimination. To our knowledge, this is one of the few STL1 cases in the literature where CI has been successfully used and followed up longitudinally.

STL1-related hearing loss often escapes neonatal hearing screening, likely due to mid-frequency preservation [1]. Otoacoustic emissions, especially distortion-product OAEs, may be reduced in excess of what pure-tone audiometry would predict [19], indicating outer hair cell dysfunction.

Conductive hearing loss is less frequent and generally associated with Eustachian tube dysfunction in children with palatal defects [1, 2, 6]. In our series, F1/P2 had cleft palate and cholesteatomatous otitis media requiring mastoidectomy, while F1/P1, from the same family and with the same variant, had normal hearing. Middle ear problems are common in individuals with cleft palate, and some may develop cholesteatoma, as observed in our patient [21, 22].

Patient F6/P1 presented with mixed hearing loss and unilateral endolymphatic sac dilation. The variant (c.3111 + 5G > A) was classified as a VUS [23]. In the absence of systemic STL1 features, its pathogenicity remains unclear. No distinctive radiological inner ear findings have been consistently reported in STL1.

Although otosclerosis has occasionally been associated with STL1 [1, 7, 19], it likely represents coincidental pathology. Hypermobile tympanic membranes and ossicular hypermobility have been reported [19, 24, 25], potentially due to abnormal type II collagen affecting middle ear structures.

Vestibular involvement in STL1 is poorly understood. One of our patients (F2/P1) presented with benign paroxysmal positional vertigo, not previously reported in STL1 but documented in other genetic hearing loss syndromes [26]. Further studies are needed to clarify whether vestibular dysfunction is part of the STL1 phenotype.

Although this study focuses on auditory findings, STL1 is a multisystem disorder. Ocular and musculoskeletal manifestations must be actively sought to ensure early diagnosis and appropriate multidisciplinary management.

The main limitations of our study are its descriptive, retrospective nature and small sample size. However, given the scarcity of published case series with detailed audiological follow-up in STL1, our findings contribute meaningful data to the existing literature.

## Conclusions

The auditory phenotype associated with mutations in the COL2A1 gene (STL1) is highly variable, with some patients not developing hearing loss, while others present with conductive hearing loss, typically secondary to Eustachian tube problems, as well as sensorineural hearing loss. The severity of hearing loss varies, ranging from mild to severe/profound cases that may require cochlear implants (CI). The response to CI in the only case that required it in our study was very favorable in terms of language comprehension.

Our results highlight the importance of personalized diagnosis, monitoring, and treatment due to the phenotypic variability of STL1. Furthermore, we emphasize the need for audiological evaluation in all patients diagnosed with STL1, allowing for the early detection of hearing loss and the timely initiation of appropriate therapeutic interventions.

## Supplementary Information

Below is the link to the electronic supplementary material.

Supplementary file1 (DOCX 14 KB)

## Data Availability

Data sharing not applicable to this article as no datasets were generated or analysed during the current study.
