# Supplementary material for: Characteristics of Hearing Loss in Patients with COL2A1 Gene Variants (Sticker Syndrome Type 1)
Source: Indian J Otolaryngol Head Neck Surg. 2025 Jun 12;77(8):3091–8. doi: 10.1007/s12070-025-05638-7 (PMC12297190; doi:10.1007/s12070-025-05638-7)
Supplement: Supplementary file 1 — Supplementary file1 (DOCX 14 KB) [file 12070_2025_5638_MOESM1_ESM.docx]

**SUPPLEMENTARY MATERIAL**

Gene panels included in NGS. The methodology of the external laboratory is described in Cabanillas et al. (34).

**Laboratorio externo (2018-2019)**

**Genes: 229**

ABHD12, ACTB, ACTG1, ADGRV1, AIFM1, ALMS1, AMMECR1, ANKH, AP1S1, ATP1A3, ATP6V0A4, ATP6V1B1, BCAP31, BCS1L, BRAF, BSND, CABP2, CACNA1D, CCDC50, CDH23, EACAM16, CHD7, CIB2, CISD2, CLCNKA, CLCNKB, CLDN14, CLPP, CLRN1, COCH, COL2A1, COL4A3, COL4A4, COL4A5, COL4A6, COL9A1, COL9A3, COL11A1, COL11A2, COLEC11, DCAF17, DDX11, DIABLO, DIAPH1, DNMT1, ECHS1, EDN3, EDNRB, EPS8L2, ESPN, ESRRB, EYA1, EYA4, FGF3, FGFR3, FTO, GATA3, GIPC3, GJB2, GJB3, GJB6, GPSM2, GRHL2, GRXCR1, GSDME, HARS1, HARS2, HGF, HOMER2, HOXA1, HOXB1, HSD17B4, ILDR1, KARS1, KCNE1, KCNJ10, KCNQ1, KCNQ4, LARS2, LHFPL5, LHX3, LOXHD1, LRP2, LRTOMT, MARVELD2, MASP1, MIR96, MITF, MSRB3, MT-CO1, MT-RNR1, MT-TH, MT-TK, MT-TL1, MT-TS1, MYH9, MYH14, MYO3A, MYO6, MYO7A, MYO15A, NARS2, NDP, NLRP3, OPA1, OSBPL2, OTOA, OTOF, OTOG, OTOGL, P2RX2, PAX3, PCDH15, PDZD7, PEX1, PEX2, PEX3, PEX5, PEX6, PEX26, PJVK, POGZ, POU3F4, POU4F3, PRPS1, PTPN11, PTPRQ, RAF1, RDX, RMND1, SALL1, SERAC1, SERPINB6, SIX1, SLC17A8, SLC19A2, SLC26A4, SLC33A1, SLC52A2, SLC52A3, SLITRK6, SMPX, SNAI2, SOX10, SPATA5, STRC, SYNE4, TBC1D24, TECTA, TIMM8A, TJP2, TMC1, TMEM132E, TMIE, TMPRSS3, TPRN, TRIOBP, TRPV4, TSPEAR, USH1C, USH1G, USH2A, WFS1, WHRN, XYLT2, ADCY1, AP3D1, ATP2B2, ATP6V1B2, BDP1, CCS, CD151, CD164, CDC14A, CLIC5, COL9A2, COQ6, CRYM, DCDC2, DIAPH3, DSPP, ELMOD3, EPS8, ERAL1, EXOSC2, FBLN1, FGFR1, FGFR2, FOXI1, GRXCR2, GSTP1, GTF2IRD1, HMX2, HMX3, KITLG, MAF, MAFB, MARS2, MCM2, MT-CO3, MT-TE, MT-TS2, NDUFA13, NFIX, PANX1, PMP22, PNPT1, POLD1, PSIP1, PTPRD, RAI1, RIPOR2, ROR1, S1PR2, SEMA3E, SIX5, SLC4A11, SLC9A1, SLC22A4, SLC26A5, SLC44A4, TBL1XR1, TK2, TMPRSS5, TNC, TUBB4B, TWIST1, WBP2, YWHAH

**Panel HUMV 1 (2020-2021)**

**Genes 188**

ABHD12, ACTB, ACTG1, ADCY1, ADGRV1, AIFM1, ALMS1, ANKH, AP1S1, ATP1A3, ATP2B2, ATP6V1B1, ATP6V1B2, BCAP31, BCS1L, BDP1, BRAF, BSND, CABP2, CACNA1D, CCDC50, CDH23, CEACAM16, CHD7, CIB2, CISD2, CLCNKA, CLCNKB, CLDN14, CLPP, CLRN1, COCH, COL11A1, COL11A2, COL2A1, COL4A3, COL4A4, COL4A5, COL4A6, COL9A1, COL9A2, COL9A3, COQ6, CRYM, DCAF17, DCDC2, DDX11, GSDME,  PJVK,  DIABLO, DIAPH1, DIAPH3, DNMT1, DSPP, ECHS1, EDN3, EDNRB, ELMOD3, EPS8, EPS8L2, ESPN, ESRRB, EYA1, EYA4, RIPOR2,  FBLN1, FGF3, FGFR1, FGFR2, FGFR3, FOXI1, FTO, GATA3, GIPC3, GJB2, GJB3, GJB6, GPSM2, GRHL2, GRXCR1, GRXCR2, GTF2IRD1, HARS2, HGF, HMX2, HMX3, HOMER2, HOXA1, HOXB1, HSD17B4, ILDR1, KARS, KCNE1, KCNJ10, KCNQ1, KCNQ4, KITLG, LARS2, LHFPL5, LHX3, LOXHD1, LRP2, LRTOMT, MAF, MARS2, MARVELD2, MASP1, MCM2, MIR96, MITF, MSRB3, MYH14, MYH9, MYO15A, MYO3A, MYO6, MYO7A, NARS2, NDP, NDUFA13, NFIX, NLRP3, OPA1, OSBPL2, OTOA, OTOF, OTOG, OTOGL, P2RX2, PAX3, PCDH15, PDZD7, PEX1, PEX2, PEX26, PEX3, PEX5, PEX6, PNPT1, POU3F4, POU4F3, PRPS1, PTPN11, PTPRQ, RAF1, RDX, RMND1, SEMA3E, SERAC1, SERPINB6, SIX1, SIX5, SLC17A8, SLC19A2, SLC26A4, SLC26A5, SLC33A1, SLC4A11, SLC52A2, SLC52A3, SLC9A1, SLITRK6, SMPX, SNAI2, SOX10, SPATA5, STRC, SYNE4, TBC1D24, TECTA, TIMM8A, TJP2, TK2, TMC1, TMEM132E, TMIE, TMPRSS3, TMPRSS5, TNC, TP63, TPRN, TRIOBP, TSPEAR, USH1C, USH1G, USH2A, WFS1, WHRN y XYLT2.

**Panel HUMV 2 (2022-2023)**

**Genes: 231**

ABHD12, ACTB, ACTG1, ADCY1, ADGRV1, AIFM1, ALMS1, AMMECR1, ANKH, AP1S1, ATP1A3, ATP2B2, ATP6V0A4, ATP6V1B1, ATP6V1B2, BCAP31, BCS1L, BDP1, BRAF, BSND, BTD, CABP2, CACNA1D, CCDC50, CD164, CDC14A, CDH23, CEACAM16, CEP78, CHD7, CHSY1, CIB2, CISD2, CLCNKA, CLCNKB, CLDN14, CLDN9, CLICK5, CLPP, CLRN1, CAR, COL11A1, COL11A2, COL2A1, COL4A3, COL4A4, COL4A5, COL4A6, COL9A1, COL9A2, COL9A3, COQ6, CRYM, DCAF17, DCDC2, DDX11, DEVIL DIAPH1, DIAPH3, DLX5, DMXL2, DNMT1, DSPP, ECHS1, EDN3, EDNRB, ELMOD3, EPS8, EPS8L2, ERAL1, ESPN, ESRRB, EYA1, EYA4, FBLN1, FDXR, FGF3, FGFR1, FGFR2, FGFR3, FITM2, FOXI1, FTO, GAB1, GATA3, GIPC3, GJB2, GJB3, GJB6, GPRASP2, GPSM2, GRAP, GREB1L, GRHL2, GRXCR1, GRXCR2, GSDME, GTF2IRD1, HARS2, HGF, HMX2, HMX3, HOMER2, HOXA1, HOXA2, HOXB1, HSD17B4, ILDR1, KARS, KCNE1, KCNJ10, KCNQ1, KCNQ4, KITLG, KMT2D, LARS2, LHFPL5, LHX3, LMX1A, LOXHD1, LRP2, LRTOMT, MAF, MAN2B1, MANBA, MARS2, MARVELD2, MASP1, MCM2, MGP, MIR96, MITF, MPZL2, MSRB3, MYH14, MYH9, MYO15A, MYO3A, MYO6, MYO7A, NARS2, NDP, NDUFA13, NFIX, NLRP3, OPA1, OSBPL2, OTOA, OTOF, OTOG, OTOGL, P2RX2, PAX1, PAX3, PCDH15, PDE1C, PDZD7, PEX1, PEX2, PEX26, PEX3, PEX5, PEX6, PJVK, PLS1, PNPT1, POU3F4, POU4F3, PPIP5K2, PRPS1, PTPN11, PTPRQ, RAF1, RAI1, RDX, REST, RIPOR2, RMND1, ROR1, S1PR2, SEMA3E, SERAC1, SERPINB6, SIX1, SIX5, SLC17A8, SLC19A2, SLC26A4, SLC26A5, SLC33A1, SLC44A4, SLC4A11, SLC52A2, SLC52A3, SLC9A1, SLITRK6, SMPX, SNAI2, SOX10, SPATA5, SPNS2, STRC, SUCLA2, SYNE4, TBC1D24, TBL1X, TECTA, TIMM8A, TJP2, TK2, TMC1, TMEM126A, TMEM132E, TMIE, TMPRSS3, TMPRSS5, TNC, TP63, TPRN, TRIOBP, TRRAP, TSPEAR, TUBB4B, TWNK, USH1C, USH1G, USH2A, WBP2, WFS1, WHRN y XYLT2.
